# Modelling of mixed-mechanism stimulation for the enhancement of geothermal reservoirs

**DOI:** 10.1098/rsta.2023.0420

**Published:** 2024-08-09

**Authors:** Hau Dang-Trung, Eirik Keilegavlen, Inga Berre

**Affiliations:** ^1^ Department of Mathematics, Center for Modeling of Coupled Subsurface Dynamics, University of Bergen, Allégaten 41, 5007, Bergen, Norway

**Keywords:** geothermal energy, mixed-mechanism stimulation, fluid injection, fracture propagation and coalescence, poroelasticity, contact mechanics

## Abstract

Hydraulic stimulation is a critical process for increasing the permeability of fractured geothermal reservoirs. This technique relies on coupled hydromechanical processes induced through pressurized fluid injection into the rock formation. The injection of fluids causes poromechanical stress changes that can lead to fracture slip and shear dilation, as well as tensile fracture opening and propagation, so-called mixed-mechanism stimulation. The effective permeability of the rock is particularly enhanced when new fractures connect with pre-existing fractures. While hydraulic stimulation can significantly improve the productivity of fractured geothermal reservoirs, the process is also related to induced seismicity. Hence, understanding the coupled physics is central, for both reservoir engineering and seismic risk mitigation. This article presents a modelling approach for simulating the deformation, propagation and coalescence of fractures in porous media under the influence of anisotropic stress and fluid injection. It uses a coupled hydromechanical model for poroelastic, fractured media. Fractures are governed by contact mechanics and a fracture propagation model. For numerical solutions, we employ a two-level approach, combining a finite volume method for poroelasticity with a finite element method for fracture propagation. The study investigates the impact of injection rate, matrix permeability and stress anisotropy on stimulation outcomes.

This article is part of the theme issue ‘Induced seismicity in coupled subsurface systems’.

## 1. Introduction

Hydraulic stimulation plays a critical role in facilitating the production of geothermal energy in low-permeability igneous rocks. Its main goal is to increase reservoir permeability to achieve flow rates that are economically feasible for commercial production [1–3]. Hydraulic stimulation can be performed at different fluid pressures. High pressures exceeding the minimum principal stress are used to open and propagate hydraulic fractures, while elevated but lower pressures can cause hydro-shearing and corresponding dilation of pre-existing natural fractures as their frictional resistance to slip is exceeded.

While hydraulic stimulation is central to enhance permeability, it is also related to induced seismicity [4–9]. Under hydraulic stimulation, it is therefore necessary to manage injection-induced seismic risks. The reactivation of faults is linked to interactions between, on the one hand, pore-pressure diffusion and poroelastic stress transfer in fractured rock and, on the other hand, changes in permeability owing to shear dilation and fracture opening and propagation. While thermal and chemical effects are secondary during short-term hydraulic stimulation (days to months), induced thermal and chemical strains from fluid reinjection can contribute to fault reactivation and seismicity in the reservoir’s production phase (months to years) [10,11]. This article focuses on the coupled hydromechanical processes involved in the short-term hydraulic stimulation of fractured reservoirs.

Injection at pressures below the minimum principal stress has been shown to be an efficient mechanism for stimulating larger volumes of rock, provided the reservoir is characterized by pre-existing natural fractures and faults and high-stress anisotropy, which is typical for igneous rocks amenable to geothermal energy production. In this case, poromechanical stress changes induced by fluid injection can cause fracture slip and corresponding shear dilation owing to the sliding of rough fracture surfaces against each other. In hard rocks, the surface roughness is sufficient to maintain a significantly enhanced fracture permeability [12,13] without the need of proppants [8,9]. For injections at pressures close to and above the minimum principal stress, the deformation of pre-existing natural fractures combines with the propagation of wing cracks in the direction normal to the least principal stress [3,14–18]. When a propagating fracture reaches another pre-existing fracture, there is no pressure concentration and low tensile stress at the propagating tip; thus, propagation is arrested [19]. The pressure increase owing to injection can then extend to the newly connected fracture, potentially causing shear slip or tensile opening and the formation of new wing cracks. As a result, the development and opening of complex fracture networks, created by the coalescence of newly formed wing cracks with pre-existing fractures, enhance the permeability of the geothermal reservoir. This mechanism of hydraulic stimulation combines (i) fracture opening by shear-dilation of pre-existing fractures and (ii) propagation of fractures, and it is referred to as mixed-mechanism stimulation [11,17,20]. It should be noted that this term is not to be confused with the term mixed-mode failure, which refers to the mode of fracture propagation.

In mixed-mechanism stimulation, the interaction of the two stimulation mechanisms of shear dilation of pre-existing fractures and tensile fracture propagation induces complex dynamics, where both the fracture apertures and the geometry of the fracture network change during stimulation. These alterations are again strongly coupled to the flow in the fractured porous medium as well as to its deformation.

Numerical modelling can be employed to study the interaction between fluid flow through fractured rock and the poromechanical deformation of the rock, including fracture deformation and propagation. The complexity of the coupled processes makes it difficult to include all such effects. Simplified models that include only a subset of the processes have commonly been considered. For instance, modelling of tensile fracturing of poroelastic media caused by high injection pressure while neglecting the effects of shear slip, contact and friction has been widely reported [21–24]. Several studies have further investigated the extension of pre-existing fracture networks in porous media resulting from fluid injection. However, these studies have either neglected friction and contact mechanics at fracture interfaces [25–27] or forced fractures to propagate along pre-defined paths [15,26].

Recently, Dang-Trung *et al*. [14] proposed a new methodology to simulate fluid flow, matrix deformation, fracture slip and fracture propagation in porous media because of fluid injection [14]. The mathematical model is based on the mixed-dimensional discrete fracture matrix (MD-DFM) model that combines the explicit representation of major fractures with a continuum representation of the surrounding medium. The model uses a co-dimension-one representation of the fractures. Hence, for a two-dimensional domain, fractures are represented as one-dimensional lines, with a longitudinal parameter representing fracture apertures. The model allows for the application of fracture contact mechanics, including frictional sliding and shear-dilation of fractures and tensile fracture opening. The framework is designed as a two-level method, with local computation of fracture propagation around individual tips separated from global computations of flow and poromechanical deformation of the fractured rock. The coupling strength between the local and global models is a user-controlled parameter that allows users to balance simulation accuracy and computational cost.

This study builds on the approach proposed by Dang-Trung *et al*. [14], delving deeper into the mixed-mechanism stimulation of fractured rock subjected to anisotropic stresses. It explores how fluid injection can change the effective poroelastic stress regime, resulting in fracture slip and dilation as well as tensile fracture propagation. Extending upon the methodology established by Dang-Trung *et al*. [14], this study advances the modelling capabilities to encompass fracture coalescence. This enables a novel analysis of the mechanisms by which new and dominant flow paths are formed within the system. It also allows for investigation of the complex interaction of coupled processes and altering structure of the fracture network, including matrix flow and poroelasticity and fracture flow, aperture changes, propagation and coalescence. The study examines how stimulation outcomes are affected by the injection rate, matrix permeability and stress anisotropy.

This article is organized as follows. Section 2 presents the mathematical model for mixed-mechanism stimulation of a fractured geothermal reservoir. In §3, we describe the numerical approach used to simulate the behaviour of the reservoir under stimulation. Section 4 presents the results of several numerical test cases, which provide insights into the role of mixed-mechanism stimulation in enhancing reservoir permeability. Finally, in §5, we present our conclusions and provide remarks about the implications of our findings.

## 2. Mathematical model

This section presents the governing equations that model fluid flow and deformation in fractured porous media, including fracture contact mechanics (allowing for fracture slip, shear-dilation and opening), propagation and coalescence. These equations are the basis for developing a simulation model that accurately captures the behaviour of fractured geothermal reservoirs under mixed-mechanism stimulation. The mathematical model is similar to the model presented by Dang-Trung *et al*. [14], and it is presented in the following subsections for completeness.

### (a) Fluid flow and poroelastic deformation of the matrix and fracture

**Figure 1.**
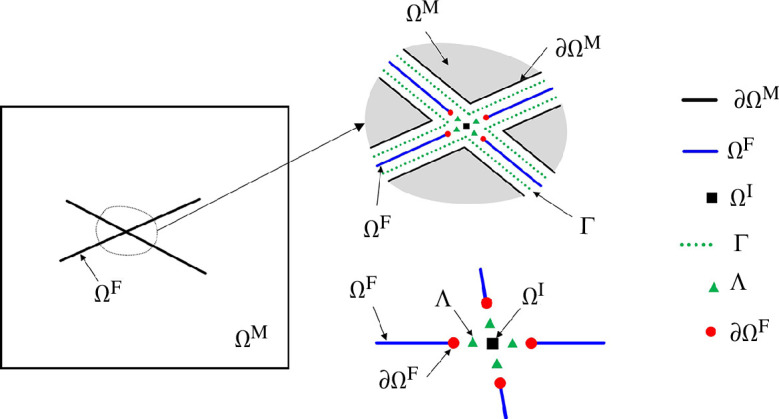
Illustration of a host medium Ω^*M*^, fractures Ω^*F*^ *,* fracture intersection Ω^*I*^ and interfaces between higher- and lower-dimensional domains, denoted by Γ and Λ, respectively. In the detailed illustrations to the right, the different domains and interfaces are separated for illustration purposes.

Based on the conceptual MD-DFM model for a two-dimensional fractured porous media, we divide the domain into the following three subdomains: a two-dimensional host medium denoted by $\Omega^{M}$ , a set of fractures represented as one-dimensional objects and denoted by $\Omega^{F}$ and fracture intersections represented as points and denoted by $\Omega^{I}$ . The boundaries of $\Omega^{M}$ and $\Omega^{F}$ are denoted by $\partial \Omega^{M}$ and $\partial \Omega^{F}$ , respectively, while Γ represents the interfaces between the host medium and fractures. When necessary, to denote the interfaces at the different sides of a fracture, we use superscripts ± on Γ . The interfaces between $\Omega^{F}$ and $\Omega^{I}$ are denoted by Λ , where the superscript i is used on Λ when necessary to denote the interface between $\Omega^{I}$ and a specific fracture indexed by i . Figure 1 provides an illustration of the model.

**Figure 2.**
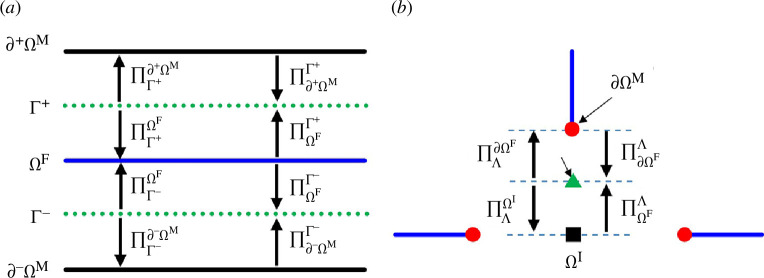
Illustration of projection operators between subdomains. (*a*) Projection operators between Ω^*M*^ and Ω^*F*^. (*b*) Projection operators between Ω^*F*^ and Ω^*I*^.

To facilitate coupling between the subdomains, projection operators $\Pi_{\left[-\right]}^{\left[-\right]}$ are introduced [28]. The illustration of these operators is given in figure 2, where the subscripts of Π indicate the origin, while the superscripts indicate the destination of the projection.

In our model, the porous media domain is considered deformable, with linear elastic, isotropic and homogeneous mechanical properties. We review the governing equations and the main variables and equations below; the other parameters are given in table 1.

**Table 1.** Parameters used in the governing equations.

notation	description	notation	description
C	stiffness matrix	$c_{p}$	fluid compressibility
*φ*	matrix porosity	μ	fluid viscosity
$\kappa_{xx}$ , $\kappa_{yy}$	permeability of the porous matrix	b	body forces
ζ	inflow from the matrix to the fracture	K	bulk modulus
κ	fracture permeability	N	number of intersecting fractures around $\Omega^{I}$
$\nabla ,\nabla_{∥}$	gradient operators	tr	trace operator

Conservation of momentum is described by


(2.1) $$\nabla \cdot \sigma =b\;on\;\Omega^{M},$$

(2.2) $$\sigma =C\nabla u-\alpha pI\;on\;\Omega^{M},$$

where u,σ and p denote displacements, poroelastic stress and pore pressure. The fluid is treated as a single phase, slightly compressible. In $\Omega^{M}$ , conservation of fluid mass is described as


(2.3) $$\alpha \frac{\partial \left(\nabla \cdot u\right)}{\partial t}+\left(\phi c_{p}+\frac{\alpha -\phi }{K}\right)\frac{\partial p}{\partial t}+\nabla \cdot q=q^{0}\;on\;\Omega^{M},$$

where $q_{0}$ is the source term, while q denotes the flux, given by


(2.4) $$q=-\frac{1}{\mu }\left[\begin{array}{cc} \kappa_{xx} & 0\\ 0 & \kappa_{yy} \end{array}\right]▽p\;on\;\Omega^{M}.$$

Here, the permeability can be heterogeneous. Conservation of mass in the fractures is governed by


(2.5) $$\frac{\partial a}{\partial t}+ac_{p}\frac{\partial p_{F}}{\partial t}+\nabla_{∥}\cdot q_{F}-\Pi_{\Gamma^{+}}^{\Omega^{F}}\lambda^{+}-\Pi_{\Gamma^{-}}^{\Omega^{F}}\lambda^{-}=q_{F}^{0}\;on\;\Omega^{F},$$

with pressure denoted by $p_{F}$ , $q_{F}^{0}$ representing source terms, and the fluid flux given by


(2.6) $$q_{F}=-\frac{\kappa a}{\mu }\nabla_{∥}p_{F}\;on\;\Omega^{F}.$$

The terms $\lambda^{\pm }$ are variables that represent the flux from the matrix to the fracture at each side of the fracture. The fracture aperture a is a function determined based on the residual aperture and normal displacement jump, such that


(2.7) $$a=a_{0}+⟦u{⟧}_{n}\;on\;\;\Omega^{F},$$

where $a_{0}$ denotes the residual aperture in the undeformed state, and ${⟦u⟧}_{n}$ represents the displacement jump in the normal direction over $\Omega^{F}$ , in which the displacement jump is defined by


(2.8) $$⟦u⟧=u{|}_{\Gamma^{-}}-\;u{|}_{\Gamma^{+}}\;on\;\Omega^{F},$$

where Γ is the interface between $\Omega^{M}$ and $\Omega^{F}$ . Finally, conservation of mass in the intersection is modelled by


(2.9) $$\frac{\partial \left(a_{I}^{2}\right)}{\partial t}+a_{I}^{2}c_{p}\frac{\partial p_{I}}{\partial t}-\sum_{i\;=1}^{N}\Pi_{\Lambda^{i}}^{\Omega^{I}}\eta_{i}=q_{I}^{0}\;on\;\Omega^{I}.$$

Here, the aperture, $a_{I}$ , is taken to be the average of the apertures of the intersecting fractures, while $q_{I}^{0}$ represents the source in the intersection. The term $\eta_{i}$ is a variable that represents the flux from fracture *i* to $\Omega^{I}$ , and N is the number of intersecting fractures that meet in $\Omega^{I}$ .

To fully represent the physical system, it is necessary to incorporate the coupling between subdomains into the mathematical model. First, the coupling between $\Omega^{M}$ and $\Omega^{F}$ is defined by


(2.10) $$q\cdot n{|}_{\partial^{\pm }\Omega^{M}}=\Pi_{\Gamma^{\pm }}^{\partial^{\pm }\Omega^{M}}\lambda^{\pm }\;on\;\partial \Omega^{M},$$

(2.11) $$\lambda^{\pm }=-\frac{\kappa }{\mu }\left(\frac{\Pi_{\Omega^{F}}^{\Gamma^{\pm }}p_{F}-\Pi_{\partial^{\pm }\Omega^{M}}^{\Gamma^{\pm }}\;tr^{\pm }p}{a/2}\right)\;on\;\Gamma^{\pm },$$

where ${\left.n\right|}_{\partial^{\pm }\Omega^{M}}$ is the outer normal of $\partial^{\pm }\Omega^{M}$ . Equation (2.10) indicates the balance of flux between the matrix and fracture. The coupling between $\Omega^{F}$ and $\Omega^{I}$ is given by


(2.12) $$q_{F}\cdot n{|}_{\partial \Omega_{i}^{F}}=\Pi_{\Lambda^{i}}^{\Omega^{F}}\eta_{i}\;on\;\partial \Omega_{i}^{F},$$

(2.13) $$\eta_{i}=-\frac{\kappa a_{I}}{\mu }\left(\frac{\Pi_{\Omega^{I}}^{\Lambda^{i}}p_{I}-\Pi_{\partial \Omega_{i}^{F}}^{\Lambda^{i}}p_{F}}{a_{I}/2}\right)\;on\;\Lambda_{i},$$

where the unit vector $n{|}_{\partial \Omega_{i}^{F}}$ is tangential to $\Omega^{F}$ and points from the fracture to $\Omega^{I}$ . The governing equations presented here are comprehensive, as they describe the mechanisms operating in each subdomain and consider their interactions.

### (b) Fracture contact mechanics

In the context of hydromechanical coupled processes, fractures are assumed to be in one of the following three mechanical states: closed and sticking (with no shear displacement) closed and slipping or open. The interactions between the fracture surfaces are governed by fracture contact mechanics. It should be noted that open and closed refer to the mechanical state of the fracture in terms of the fracture surfaces being in contact or not. In a mechanically closed state, the fracture can still be hydraulically open with a hydraulic aperture that can be enhanced by the fracture sliding and corresponding dilation.

In the following, the fracture contact mechanics model is considered independently in the normal and tangential directions. We define n as the normal vector of the fracture pointing from $\Gamma^{+}$ to $\Gamma^{-}$ , and define the contact traction f according to the direction of n .

The normal opening of the fracture is governed by a non-penetration condition written in Karush–Kuhn–Tucker form [29] as


(2.14) $$⟦u{⟧}_{n}-g\geq 0,\;f_{n}\leq 0,\;\left(⟦u{⟧}_{n}-g\right)f_{n}=0\;on\;\Omega^{F}.$$

Here, $f_{n}$ represents the contact traction in the normal direction, and g is a gap function defined by


(2.15) $$g=-tan(\psi )|⟦u{⟧}_{\tau }|\;on\;\Omega^{F},$$

where ψ is the dilation angle and ${⟦u⟧}_{\tau }$ is the displacement jump in the tangential direction. The gap function in equation (2.15) accounts for the dilation of the fracture resulting from tangential slip while maintaining contact between the fracture surfaces. This feature enables the enhancement of permeability in the fracture owing to shear dilation.

The tangential motion of the fracture is modelled as a frictional contact problem given by


(2.16) $$\begin{array}{l} \left|f_{\tau }\right|\leq -\mu_{s}f_{n}\\ {\left|f\right|}_{\tau }<-\mu_{s}f_{n}\to ⟦\overset{˙}{u}{⟧}_{\tau }=0\\ \left|f_{\tau }\right|=-\mu_{s}f_{n}\to \exists \varepsilon \in R,\;f_{\tau }=-\varepsilon ⟦\overset{˙}{u}{⟧}_{\tau }\;\;\; \end{array}\;\;\;\;\;\;\;\;\;\;\;\;on\;\Omega^{F},$$

where $\mu_{s}$ represents the friction coefficient and $\dot{u}$ is the derivative of u with respect to time. The contact traction in the tangential direction, $f_{\tau }$ , contains directional information and is, therefore, a vector despite the fracture being one-dimensional. Traction on the fracture surfaces balances the pressure in the fracture by Newton’s third law and can be expressed as


(2.17) $$\Pi_{\partial^{+}\Omega^{M}}^{\Gamma^{+}}\left(\sigma \cdot n{|}_{\partial^{+}\Omega^{F}}\right)=\Pi_{\Omega^{F}}^{\Gamma^{+}}\left(f-\alpha_{f}p_{F}n\right)\;\;\;\;\;\;\;\;\;\;\;\;\;\;\;\;\;\;\;\;\;\;on\;\Gamma^{+},$$

(2.18) $$\Pi_{\partial^{-}\Omega^{M}}^{\Gamma^{-}}\left(\sigma \cdot n{|}_{\partial^{-}\Omega^{F}}\right)=-\Pi_{\Omega^{F}}^{\Gamma^{-}}\left(f-\alpha_{f}p_{F}n\right)\;on\;\Gamma^{-},$$

with equations (2.17) and (2.18) indicating that the traction on the fracture surfaces is caused not only by the matrix deformation and pressure but also by pressure in the fracture.

To explain the governing equations (2.14) and (2.18) further, we can first consider the situation of a fracture being closed (i.e. contact between the fracture surfaces). In this case, $⟦u{⟧}_{n}=g$ and $f_{n}\leq 0$ . Closed fractures can either be in the state of sticking, in which $\left|f_{\tau }\right|<-\mu_{s}f_{n}$ , or they can be sliding, in which $\left|f_{\tau }\right|=-\mu_{s}f_{n}$ . The third state is the fracture being open. In this case, $f_{n}=\left|f_{\tau }\right|=0$ . Furthermore, as an example, we see from equations (2.17) and (2.18), that if a fracture is closed and sticking, an increase in fracture pressure will increase $f_{n}$ and thereby enhance its possibility to slip. A further increase in pressure may lead to $f_{n}$ becoming zero, in which case the fracture surfaces are no longer in contact and the fracture is mechanically open.

### (c) Fracture propagation

We combine the maximum tangential stress criterion [30] and Paris’s law [31] to determine the onset of fracture propagation as well as the propagation direction and length. The maximum tangential stress criterion assumes that a fracture propagates when the maximum tangential stress in the process zone around a fracture tip exceeds a critical value defined as


(2.19) $$K_{I}\cos^{3}\frac{\theta }{2}-\frac{3}{2}K_{II}\cos\frac{\theta }{2}\sin\theta \geq K_{IC}\;.$$

The direction of propagation is that of the maximum tangential stress given by


(2.20) $$\theta =2\tan^{-1}\left(\frac{K_{I}}{4K_{II}}\pm \frac{1}{4}\sqrt{{\left(\frac{K_{I}}{K_{II}}\right)}^{2}+8}\right)\;,$$

(2.21) $$K_{II}\left(\sin\frac{\theta }{2}+9\sin\frac{3\theta }{2}\right)<K_{I}\left(\cos\frac{\theta }{2}+3\cos\frac{3\theta }{2}\right)\;,$$

where $K_{I}$ and $K_{II}$ are the stress intensity factors (SIFs) calculated by using the nodal displacement correlation technique [32] in conjunction with quarter-point elements around the crack tip [33,34]. If more than one crack grows simultaneously, then the tips in the fracture with higher energy advance farther than the others, with a distribution given by the Paris-type law [31]


(2.22) $$l_{adv}^{i}=l_{\max}{\left(\frac{G_{i}}{\max\left(G_{i}\right)}\right)}^{0.35},$$

where $l_{adv}^{i}$ and $G_{i}$ are the propagation length and energy release for tip *i*, respectively [35]. By equation (2.22), the increment for each tip is limited by a preset value, $l_{\max}$ .

A propagating fracture may reach and coalesce with another fracture in a T-type connection. This leads to the formation of a new intersection point that is added to $\Omega_{I}$ and new connections between the merged fractures and the intersection.

## 3. Discretization method

In this section, we describe a numerical approach for discretizing the mathematical model presented in §2. As the model depends on both space and time variables, both variables must be discretized. Time discretization can be achieved using the backward Euler method, while spatial derivatives are dealt with by the two-level simulation approach recently proposed by Dang-Trung *et al*. [14].

The motivation for using the two-level simulation approach is to balance computational cost and simulation accuracy. Specifically, poroelastic deformations with fracture contact mechanics, but without fracture propagation, are assumed to be quasi-static and are treated using a relatively coarse grid. In contrast, a locally refined grid around the fracture tip is needed to accurately capture the interaction between fracture propagation and local stress variations. If a fracture propagates and exceeds a certain threshold length, the geometry of the fracture network and the solution are updated in the coarse-level domain for the next time step. A brief description of this approach is provided below; for more information, we refer to Dang-Trung *et al*. [14].

**Figure 3.**
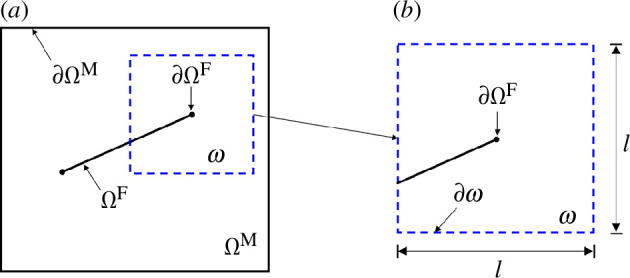
Illustration of a fracture, *Ω* ^*F*^, (*a*) and a fine-level domain, *ω*, (*b*) adapted from Dang-Trung *et al*. [14].

The computational domain is divided into a coarse-level domain that matches the entire domain and smaller, local fine-level domains with size *l* that surround the fracture tips. The coarse-level and fine-level domains are denoted by $\Omega^{M}$ and ω , respectively, as illustrated in figure 3. These domains are discretized using triangular cells with grid sizes ΔH and Δh for $\Omega^{M}$ and ω , respectively. The grids conform to the fractures so that fracture surfaces coincide with grid faces. An adaptive remeshing technique is employed to represent fracture paths in the grids and avoid excessive computational costs while ensuring an accurate numerical solution at the relevant scale of the model [35]. This technique uses finer cells around fracture tips in both coarse-level and fine-level grids to capture the details of fracture propagation. Additionally, to ensure the stability of the simulation, the resolution of the fine-level grid is set to be finer than that of the coarse-level grid, i.e. $∆h=\varepsilon_{m}∆H$ with $\varepsilon_{m}\leq 1$ .

When the local fine-level domains contain only one fracture segment, the coarse-level and fine-level domains are defined differently. However, if a local fine-level domain associated with a fracture contains segments of neighbouring fractures, the fine-level domain is set to be identical to the full domain, as the current implementation is restricted to local fine-level domains with a single fracture segment.

### (a) Two-level discretization

The poroelastic deformation model presented in §2(a)and 2(b) is discretized based on the coarse-level grid. Specifically, the governing equations in §2(a) are discretized using a finite volume approach with a multi-point flux approximation and a multi-point stress approximation [36,37], while the fracture contact mechanics model presented in §2(b) is discretized by an active set method [29,38,39]. The solution at this level provides the deformation and fluid pressure in the poroelastic domain and determines fracture mechanical behaviour, whether the fracture is open, closed and sticking or closed and slipping.

The fine-level domain is responsible for evaluating fracture propagation at each time step. To do this, we combine equations (2.1) and (2.2) and assume that the fine-level domain behaves similarly to a linearly elastic medium governed by:


(3.1) $$\nabla \cdot \left(c\nabla_{s}u_{l}\right)+b=0,$$

where $u_{l}$ is the deformation in the fine-level domain, $b=-\nabla \cdot \left(\alpha pI\right)$ is the body force caused by pressure from the coarse-level domain and c is the stiffness tensor. The boundary conditions for the fine-level problem, i.e. defined at ∂ω , are set according to the coarse-level state. To solve equation (3.1), we use a combination of the $P_{2}$ finite element method and quarter-point elements to accommodate the stress singularity at the fracture tip [32,33]. This solution obtained is used to compute SIFs and determine whether a fracture will propagate and, if so, where and how far it will go, as described in §2(c). The maximum increment of fracture is set to the fine-level grid size, i.e. $l_{max}=\Delta h$ .

### (b) Coupling between coarse-level and fine-level solutions

To establish the numerical coupling between the coarse-level and fine-level domains, it is necessary to project the displacements from the coarse-level to the fine-level domain boundaries and compress the fine-level updates to the fracture geometry in the coarse-level grid. These projections can be achieved using three mapping processes: cell centre to cell centre, node to node and cell centre to node [14]. Additionally, updating the coarse-level fracture path is necessary if the propagation length in the fine-level domain is sufficient to cause a considerable change in the coarse-level grid. To accomplish this, we denote $\left|∆\omega^{F}\right|$ as the total propagation length in a fine-level domain. If $\left|∆\omega^{F}\right|$ exceeds $\varepsilon_{p}∆H$ , with $\varepsilon_{p}$ being a propagation factor, the coarse-level fracture is extended using a linear approximation of $∆\omega^{F}$ , and the coarse-level grid is updated.

### (c) Fracture coalescence

**Figure 4.**
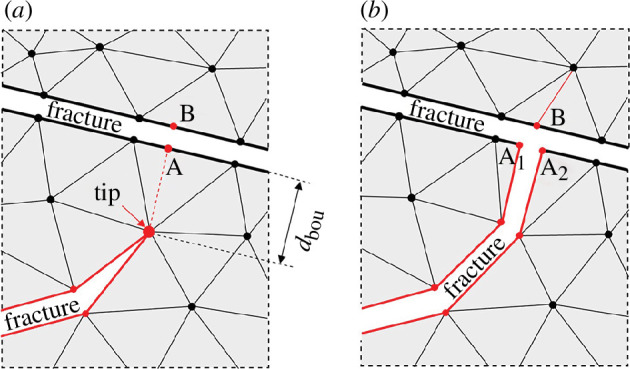
The T-type intersection between fractures or between a fracture and boundary. The fracture is widened for illustration purposes. (*a*) Determination of intersection point (A). (*b*) Fractures joined by a T-type connection.

This paper models the fracture intersection by a T-type connection. As illustrated in figure 4*a*, when the distance between a propagating crack tip and another fracture is less than the grid size around the tip, the two fractures are assumed to be connected. A connection point is identified by projecting the fracture tip onto the boundary, resulting in point A. Point B is then defined as the projection of point A to the opposite side of the connected fracture boundary. Finally, the tip of the propagating fracture is split at point A to create a T-type connection, as depicted in figure 4*b*.

## 4. Results

The accuracy of simulations of fracture propagation and fluid flow in the fractured porous media domain was partially verified in previous studies [14,28,35,40–42]. The numerical examples in this section aim to show the ability of the proposed model to simulate complex problems, such as multiple fractures deforming, propagating and connecting in a medium with heterogeneous permeability. The source code for the following simulations is open-sourced and can be found by referring to [43].

This section presents four numerical examples to investigate the effects of the fluid injection rate, principal stress, permeability and fracture network on mixed-mechanism stimulation for a fractured low-permeability porous medium. The idealized setups are chosen to be representative of settings that could be found in geothermal reservoirs in igneous rocks. Given the limitation of our resources, a relatively small domain with a few pre-existing fractures is considered. For all cases, the coordinates of the tips, the material and the simulation parameters are given in tables 2–4 respectively.

**Table 2.** Tips coordinates (units: m).

tip	x	y	Tip	x	y
A	1.00	1.15	B	1.00	0.85
C	0.85	0.97	D	1.15	1.03
E	0.65	1.10	F	0.65	0.90
G	1.40	1.06	H	1.28	0.94

**Table 3.** Material properties.

parameter	definition	value
Ε	Young’s modulus	40.0GPa
ν	Poisson’s ratio	0.2
$K_{IC}$	fracture toughness	$1.0\;MPa\cdot m^{1/2}$
α	Biot’s coefficient in the matrix	0.8
φ	material porosity	0.01
$c_{p}$	fluid compressibility	$4.4\times 10^{-10}\;Pa^{-1}$
μ	viscosity	$1.0\times 10^{-4}\;Pa\cdot s$
$\mu_{s}$	friction coefficient	0.5
ψ	dilation angle	$1.0^{o}$
$a^{0}$	initial aperture	1.0 mm

**Table 4.** Simulation parameters.

parameter	definition	value
$L_{x}=L_{y}$	coarse-level domain size	2.0 m
l	fine-level domain size	0.1 m
ΔH	coarse-level grid size	0.02 m
∆h	fine-level grid size	0.01 m
$\varepsilon_{m}$	ratio between coarse-grid and fine-grid sizes	0.5
$\varepsilon_{p}$	propagation factor	0.5
∆t	time step	0.5 min

### (a) Effect of principal stress direction

**Figure 5.**
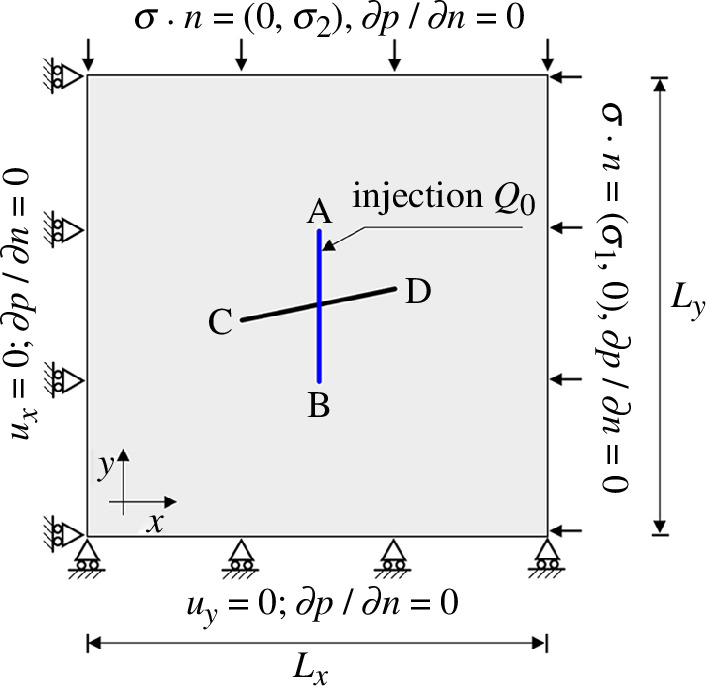
The geometry and boundary conditions of model 1.

**Figure 6.**
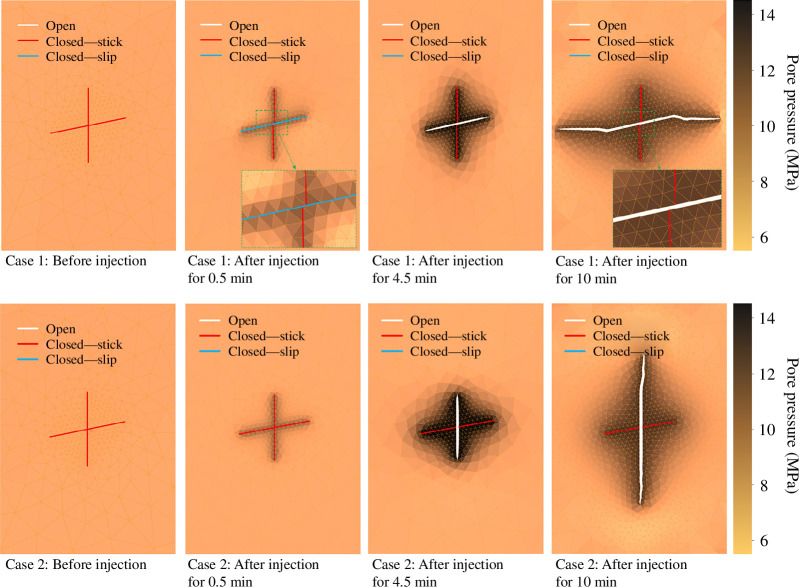
Fracture propagation and pressure evolution in a 2D porous media during fluid injection into a pre-existing fracture for two cases that differ in the background principal stress. The solid white lines indicate opening fractures, while the solid red lines indicate closed fractures. The colour bar represents pore pressure in Mpa.

First, the effect of the background principal stress on fracture propagation is investigated. As illustrated in figure 5, we consider a 2D domain containing two intersecting fractures and the boundary conditions prescribed in this figure (model 1). We assume that the matrix permeability of the domain is isotropic and homogeneous, given by $\kappa_{xx}=\kappa_{yy}=5.0\times 10^{-20}\;m^{2}$ . The fractured porous medium is subject to a stress state imposed orthogonally to the domain. Fluid is injected into the vertical fracture continuously at a constant rate of $Q_{0}=1\times 10^{-7}\;m^{2}/s$ . Two stress scenarios are considered. For case 1, $\sigma_{1}=2\sigma_{2}=20$ MPa, and for case 2, $2\sigma_{1}=\sigma_{2}=20$ MPa. The propagation of the fractures, presented by solid lines, and the fluid flow, described by colour, are shown in figure 6.

In both scenarios, pre-existing fractures are closed before fluid is injected owing to compressive stress and friction at the fracture interfaces. Depending on the stress regime, the injection can lead to slip in pre-existing fractures. After 0.5 min of injection, in case 1, the fracture, which is nearly parallel to the direction of maximum stress, slips. At the same time, in case 2, both fractures remain undeformed, i.e. in the stick mode. In both cases, the vertical fracture is closed and remains in stick mode under compressive stress.

It is well known that fractures propagate towards the direction of maximum principal stress. In case 1, the low injection rate of the fluid does not provide sufficient pressure to induce tensile propagation of the vertical fracture where fluid is injected. However, it does cause shear slip and dilation of the nearly horizontal crossing fracture early in the stimulation process. Continued injection results in wing cracks that appear after 4.5 min and propagate in the direction of the maximum principal stress. Thus, this test case demonstrates an example of mixed-mechanism stimulation, where both shear slip and tensile fracture propagation occur during the stimulation. In case 2, continued fluid injection combined with the shifted stress anisotropy causes the vertical fracture in which the fluid is injected to open. Shear slip does not occur in this case, and tensile propagation of the vertical fracture initiates after 7 min of injection once the fluid pressure has built up sufficiently.

The simulation also displays the state of fractures, whether they are closed in stick mode, closed in slip mode or open. A red line indicates a section of a fracture in stick mode, while a light-blue line indicates a section in slip mode. A section of a fracture in open mode is indicated by a solid white line.

### (b) Effect of matrix permeability

**Figure 7.**
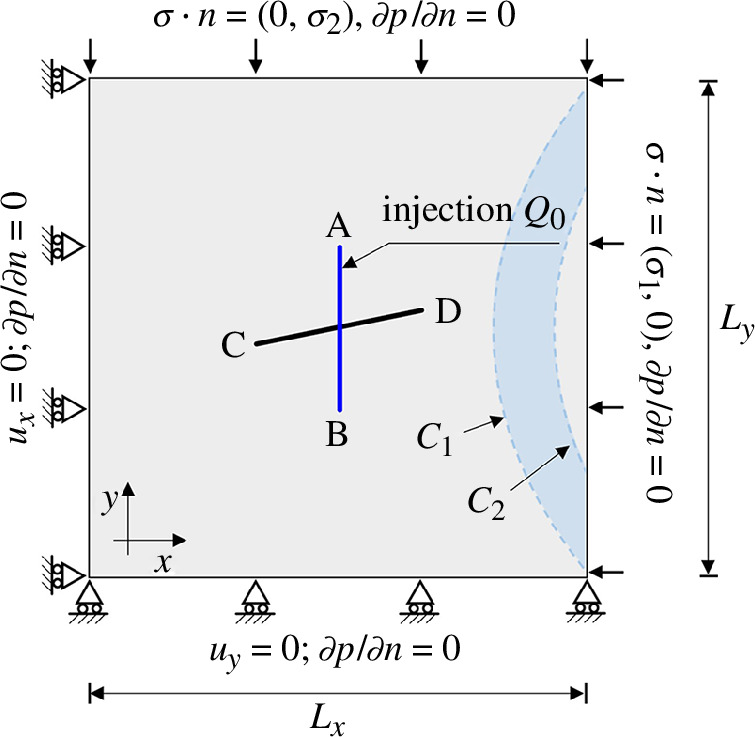
The geometry and boundary conditions of model 2.

**Figure 8.**
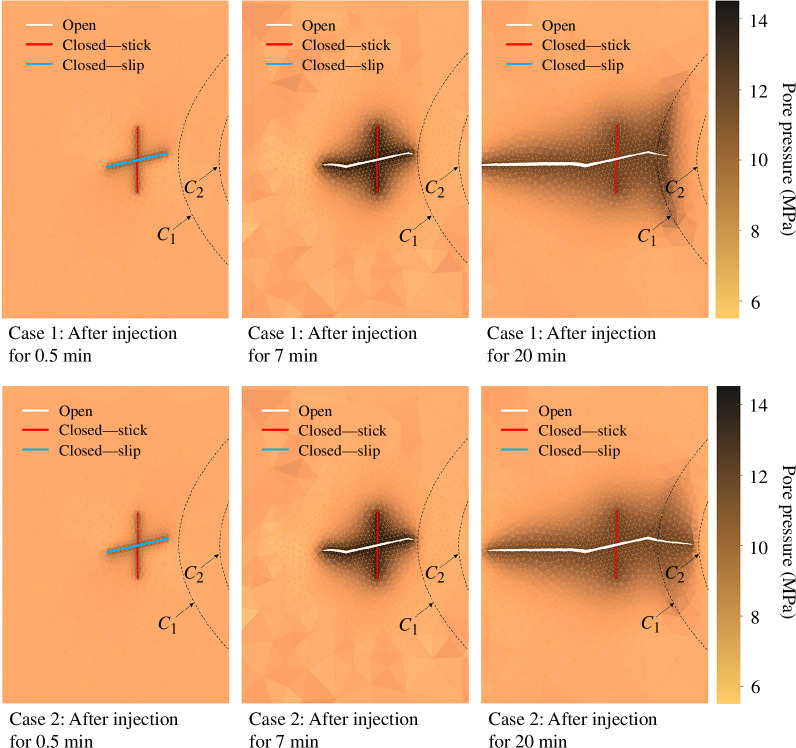
Fracture propagation and pressure evolution in a two-dimensional porous medium during fluid injection into a pre-existing fracture for two cases. For case 1, the permeability in the principal *y*-direction in the region between the curves *c*_1_ and *c*_2_ is higher than it is for case 2. The solid white lines indicate open fractures, while the solid red lines indicate closed fractures. The colour bar represents pore pressure in Mpa.

This study examines the influence of matrix permeability on fracture propagation within a 2D domain with heterogeneous permeability (model 2). Two distinct permeability regions are investigated, as illustrated in figure 7. Region 1 is bounded by the curves $C_{1}:x-{\left(y-1\right)}^{2}-1.2=0$ , $C_{2}:x-{\left(y-1\right)}^{2}-1.4=0$ and the right boundary, while region 2 is the remainder. The permeability in region 2 is homogeneous and isotropic with values of $\kappa_{xx}=\kappa_{yy}=5\times 10^{-20}\;m^{2}$ . Two simulation cases are conducted, depending on the permeability of region 1. For case 1, $\kappa_{xx}=5\times 10^{-20}\;m^{2}$ and $\kappa_{yy}=5\times 10^{-18}\;m^{2}$ , while for case 2, $\kappa_{xx}=5\times 10^{-20}\;m^{2}$ and $\kappa_{yy}=5\times 10^{-19}\;m^{2}$ . Additional parameters used for the simulations are $\sigma_{1}=2\sigma_{2}=20$ MPa and $Q_{0}=1\times 10^{-7}\;m^{2}/s$ . The propagation of the fractures and the fluid flow are shown in figure 8.

The presence of a highly permeable area inhibits fracture growth by preventing fluid pressure from building up sufficiently owing to fluid leakage into the matrix. For both case 1 and case 2, like the model 1 case 1 in §4a, the principal stress scenario and fluid injection first induce slip of the nearly horizontal fracture. The appearance of wing cracks happens after 5.5 min of injection for both cases. The wing cracks then propagate to opposite sides and contact with the area of higher permeability after 7 min. This contact causes further fluid leakage from the fracture to the matrix, which slows the fracture growth rate. Additionally, the tip in contact with the higher-permeability region propagates much more slowly than the other. Both propagate in the direction of the maximum principal stress. In both cases studied, the fractures could not propagate through the higher-permeability region. This example clearly illustrates the sensitivity of fracture propagation to matrix permeability and demonstrates that simulation tools that do not capture the fluid flow between fracture and matrix and cannot accurately represent the propagation process.

### (c) Effect of injection rate

**Figure 9.**
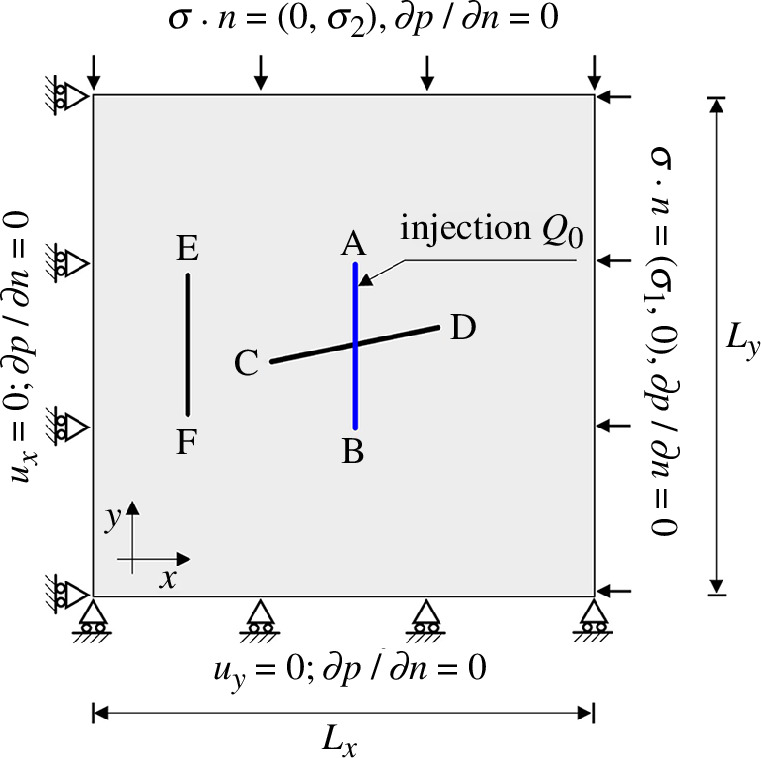
The geometry and boundary conditions of model 3.

**Figure 10.**
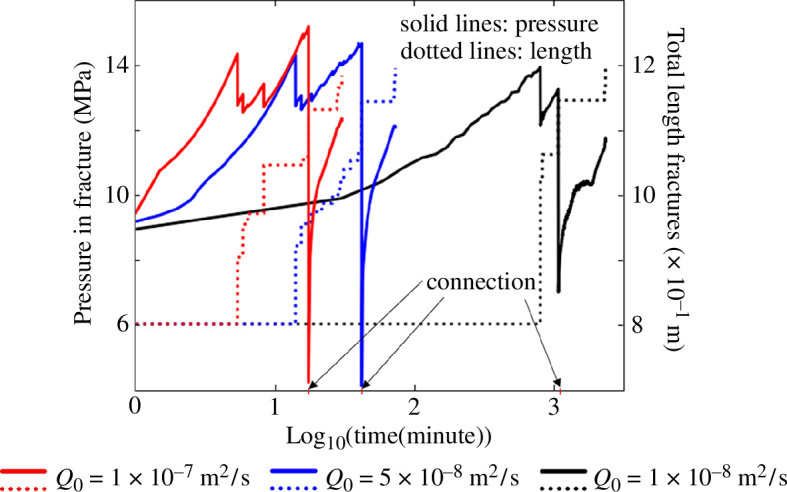
Effect of fluid injection rate on pressure at the injection point and total fracture growth.

This example investigates the effect of the injection rate on the fracture propagation for a case where the propagating fracture coalesces with a pre-existing fracture. Figure 9 illustrates the considered two-dimensional fractured domain containing three fractures with boundary conditions described in the figure (model 3). We assume that the permeability is isotropic and homogeneous, given by $\kappa_{xx}=\kappa_{yy}=5.0\times 10^{-20}\;m^{2}$ . The principal stress is given by $\sigma_{1}=2\sigma_{2}=20$ MPa. Various injection rates are studied, and the effect on fracture growth and pressure in the fracture is shown in figure 10.

The results shown in this example indicate that an increase in the injection rate leads to faster fracture propagation, and the propagation speed is nonlinearly dependent on the injection rate. As illustrated in figure 10, wing cracks initiate after 4.5 min for an injection rate of $Q_{0}=1\times 10^{-7}\;m^{2}/s$ , whereas it takes up to 870 min for an injection rate of $Q_{0}=1\times 10^{-8}\;m^{2}/s$ . This indicates that increasing the injection rate by a factor of 10 can accelerate the expansion of the fracture network by up to 200 times. However, if the injection rate is too low, then no fracture deformation occurs during the simulation.

### (d) Interaction with pre-existing fractures

**Figure 11.**
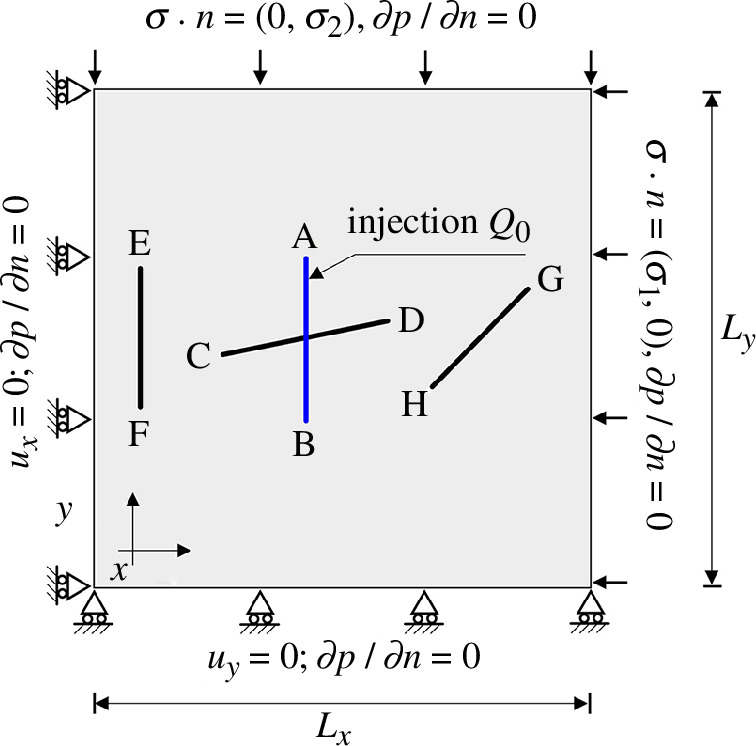
The geometry and boundary conditions of model 4.

Finally, we investigate the influence of the location of pre-existing fractures on the expansion of the fracture network. The model geometry and boundary conditions are shown in figure 11 (model 4). The matrix permeability in this example is assumed to be isotropic and homogeneous, i.e. $\kappa_{xx}=\kappa_{yy}=5.0\times 10^{-20}\;m^{2}$ . The principal stress is given by $\sigma_{1}=2\sigma_{2}=20$ MPa. The injection rate is $Q_{0}=2\times 10^{-7}\;m^{2}/s$ .

**Figure 12.**
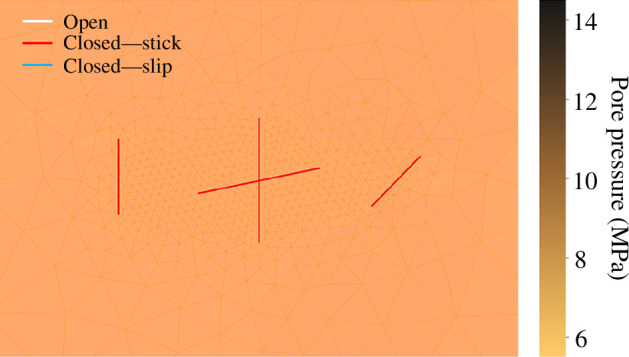
Fracture state and pressure in a two-dimensional porous medium before fluid is injected.

Prior to fluid injection, the fracture mode and pore pressure are evaluated. As illustrated in figure 12, pre-existing fractures are closed and remain in stick mode owing to compressive stress and friction at the fracture interfaces. Additionally, the pressure throughout the domain is uniform at 6.8 MPa. The result in this simulation indicates a stable condition with no fracture slip or propagation.

**Figure 13.**
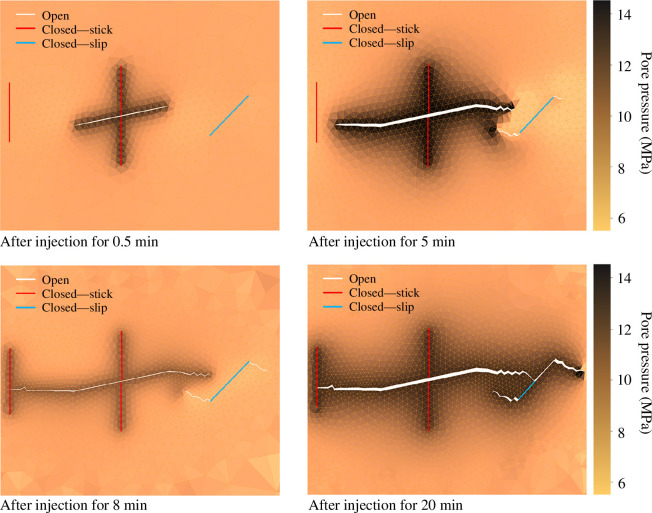
Fracture propagation and pressure evolution in a two-dimensional porous medium during fluid injection, *Q*_0_ = 2 × 10^−7^ m^2^/s, into a pre-existing fracture. The solid white lines indicate open fractures, while the solid red lines indicate closed fractures. The colour bar represents pore pressure in MPa.

Subsequently, fluid is injected into a vertical fracture, resulting in several interesting phenomena, as shown in figure 13. First, the injection has an insignificant effect on the state of the fracture where fluid is injected, as it remains under compression under the influence of the stress regime. However, the injection facilitates the opening of the horizontal fracture connected to it and leads to the propagation of this fracture. Second, owing to deformation and hydromechanical stress changes caused by fluid injection, the pre-existing fracture to the right of the domain starts to slip at an early stage of fluid injection. Eventually, small wing cracks are observed to form at the tips of this fracture. Third, there is a strong link between fracture propagation and pressure drop in the fracture. After a period of fluid injection, the pressure in the central, nearly horizontal, fracture increases sufficiently to cause tensile propagation of the fracture, which ultimately connects to the pre-existing fractures at the left and right. Each connection results in an instantaneous decrease in pore pressure, which takes time to recover through fluid injection before the fracture can resume growing. Furthermore, the expansion of the fractured network is influenced by the pre-existing fractures. During the simulation, the fracture on the right-hand side where the slip occurs continues to grow, while the fracture on the left side where compression occurs (closed in stick mode) prevents further network expansion.

## 5. Conclusions

This article describes mixed-mechanism stimulation of fractured reservoirs. The mathematical model combines poroelasticity and fracture mechanics and accounts for frictional contact mechanics and fracture propagation and coalescence. A two-level model that combines finite volume and finite element methods is used for numerical simulations. Several numerical examples are performed, and the results corroborating previous findings are as follows:

— Fluid injection at elevated pressure can induce shear slip and dilation, opening and propagation of fractures. Newly formed fractures tend to propagate in the direction of maximum principal stress. In the case of multiple connected fractures in an anisotropic stress field, the propagation of fractures depends on fracture network characteristics such as fracture orientation relative to the stress field and whether fractures are hydraulically connected to the well through other fractures.
— A more permeable bulk domain slows fracture growth by causing fluid leakage into the matrix, making hydraulic stimulations less effective in areas with higher permeability.
— The relationship between the injection rate and fracture growth speed is nonlinear, and injection at a low rate may not result in fracture expansion. In most cases, when the injection rate is low, the injection time required for a fracture to propagate is significantly longer.
— The locations of pre-existing fractures influence the expansion of a fracture network. Fractures tend to propagate in the direction of the maximum principal stress, and pre-existing fractures can facilitate or impede the development of propagating fractures.

In conclusion, this study demonstrates how advanced numerical models can be used in understanding the mechanisms behind mixed-mechanism stimulation. It shows how mixed-mechanism stimulation can significantly improve permeability by deforming and expanding a pre-existing fracture network. However, this expansion is complex and influenced by various factors, including the poroelastic stress state, material permeability, injection rate and fracture location. The simulation model proposed in this study represents an approach that is appropriate for utilization in future studies to further investigate these phenomena.

## Data Availability

The source code for the following simulations is open-sourced and it is available at Zenodo [43].
